# Temporal profiles of age-dependent changes in cytokine mRNA expression and glial cell activation after status epilepticus in postnatal rat hippocampus

**DOI:** 10.1186/1742-2094-8-29

**Published:** 2011-04-08

**Authors:** Juha T Järvelä, Francisco R Lopez-Picon, Anna Plysjuk, Saku Ruohonen, Irma E Holopainen

**Affiliations:** 1 Department of Pharmacology, Drug Development, and Therapeutics, Institute of Biomedicine, University of Turku, Itäinen Pitkäkatu 4B, FIN-20014 Turku, Finland; 2 Turku PET Center, Preclinical imaging, Tykistökatu 6A, 4th Floor, FIN-20520 Turku, Finland; 3 Medicity Research Laboratory, Tykistökatu 6A, 4th Floor, Institute of Biomedicine, University of Turku, FIN-20014 Turku, Finland

## Abstract

**Background:**

Status epilepticus (SE) is proposed to lead to an age-dependent acute activation of a repertoire of inflammatory processes, which may contribute to neuronal damage in the hippocampus. The extent and temporal profiles of activation of these processes are well known in the adult brain, but less so in the developing brain. We have now further elucidated to what extent inflammation is activated by SE by investigating the acute expression of several cytokines and subacute glial reactivity in the postnatal rat hippocampus.

**Methods:**

SE was induced by an intraperitoneal (i.p.) injection of kainic acid (KA) in 9- and 21-day-old (P9 and P21) rats. The mRNA expression of interleukin-1 beta (IL-1β), tumor necrosis factor-alpha (TNF-α), interleukin-10 (IL-10), matrix metalloproteinase-9 (MMP-9), glial-derived neurotrophic factor (GDNF), interferon gamma (IFN-γ), and transforming growth factor-beta 1 (TGF-β1) were measured from 4 h up to 3 days after KA injection with real-time quantitative PCR (qPCR). IL-1β protein expression was studied with ELISA, GFAP expression with western blotting, and microglial and astrocyte morphology with immunohistochemistry 3 days after SE.

**Results:**

SE increased mRNA expression of IL-1β, TNF-α and IL-10 mRNA in hippocampus of both P9 and P21 rats, their induction being more rapid and pronounced in P21 than in P9 rats. MMP-9 expression was augmented similarly in both age groups and GDNF expression augmented only in P21 rats, whereas neither IFN-γ nor TGF-β1 expression was induced in either age group. Microglia and astrocytes exhibited activated morphology in the hippocampus of P21 rats, but not in P9 rats 3 d after SE. Microglial activation was most pronounced in the CA1 region and also detected in the basomedial amygdala.

**Conclusion:**

Our results suggest that SE provokes an age-specific cytokine expression in the acute phase, and age-specific glial cell activation in the subacute phase as verified now in the postnatal rat hippocampus. In the juvenile hippocampus, transient increases in cytokine mRNA expression after SE, in contrast to prolonged glial reactivity and region-specific microglial activity after SE, suggest that the inflammatory response is changed from a fulminant and general initial phase to a more moderate and specific subacute response.

## Background

Status epilepticus (SE) has been shown to cause induction of the innate immunity system in brain, including hippocampus. In adult rats, several inflammatory mediators are activated in response to SE. These include upregulation of cyclooxygenase-2 (COX-2) and cytokines, e.g. interleukin-1β (IL-1β), tumor necrosis factor-α (TNF-α), and interleukin-6 (IL-6), together with morphological and functional changes in glial cells, i.e. microglia and astrocytes, from resting to active states after either pilocarpine- or kainic acid- (KA) induced seizures [1-3]. Furthermore, upon activation by seizures, cytokine (e.g. IL-1β, TNF-α, and IL-6) expression is increased in both glial cell types [4]. It has been suggested that these responses could contribute to excitotoxic neuronal damage in the hippocampus, a brain region especially susceptible to injury caused by SE [5-7].

In contrast to adult brain, SE-induced acute activation of inflammatory processes seems to be age-specifically regulated in the developing postnatal rat brain, e.g. postnatal-day 21 (P21) rats exhibit pronounced inflammatory responses (increases in COX-2 and pro-inflammatory cytokine expression, and glial cell reactivity) within 24 h after SE in hippocampus whereas this is not seen in P9 rats [6,8]. Additionally, SE-induced neuronal damage has been shown to differ according to age; younger brain being more resistant to seizure-induced damage than older brain [9,10]. In the hippocampus of juvenile rats, neuronal damage caused by a single episode of SE is localized in the CA1 or CA3 pyramidal cell regions [6,8]. Although more thoroughly studied in the adult rat brain, classical pro-inflammatory cytokines (IL-1β, TNF-α, and IL-6) have been found to be upregulated within a few hours after SE in juvenile hippocampus as well [6]. However, whether SE leads to augmented expression of other cytokines involved in inflammation has remained unaddressed in the developing hippocampus. Such factors include anti-inflammatory cytokines, matrix metalloproteinases, growth factors and interferons. The anti-inflammatory cytokine, interleukin-10 (IL-10), can suppress pro-inflammatory cytokine signalling in macrophages and microglia by inhibiting STAT-1α activation [11], and IL-10 expression is upregulated after excitotoxic lesions in P9 rat brain [12]. Matrix metalloproteinases (MMP) are known to regulate the metabolism of the pericellular environment and, in particular, MMP-9 activity is increased after KA-induced SE in the hippocampus of adult rats [13]. Furthermore, transforming growth factor-β1 (TGF-β1) has been proposed to protect neurons from rapid KA-induced excitotoxicity, possibly by inhibiting the rise in intracellular Ca^2+ ^[14], and on the other hand, to contribute to remodelling of brain after injury by regulating expression of MMP-9 in astrocytes and inducing their migration [15]. Glial cell-derived neurotrophic factor (GDNF) is a potent survival factor for several types of neurons, and its mRNA expression is increased in adult rat hippocampus 6-24 h after intraperitoneal (i.p.) KA injection [16]. In addition, interferon-γ (IFN-γ) has been suggested to have a neuroprotective function as well, since it may induce neurotrophic growth factor (NGF) production in activated astrocytes obtained from rat brain that has been lesioned by intracerebral 6-hydroxydopamine injection [17]. Also, the temporal behaviour of glial cells after SE is incompletely known in P9 and P21 rat hippocampus, since there are no *in vivo *studies at these ages in which the reactivity of glial cells has been investigated beyond 24 h after KA-induced SE.

The current study was designed to obtain more detailed information of the temporal and developmental profiles of inflammatory processes activated by SE. For this purpose, we investigated mRNA expression of the cytokines IL-1β, TNF-α, IL-10, MMP-9, GDNF, IFN-γ, and TGF-β1 with real-time quantitative PCR (qPCR) in P9 and P21 rats 4-24 h, and also in P21 rats 3 d after KA-induced SE. Additionally, IL-1β protein expression was measured 8 h after SE in P21 rats. Immunohistochemistry was used to investigate microglial and astrocyte morphology subacutely (3 days) after SE, and the observed changes were quantified by counting microglial cells with active morphology and by GFAP western blotting, respectively.

## Methods

### Experimental animals in kainic acid-induced status epilepticus

All animal experiments were conducted in accordance with the guidelines of the European Community Council Directives 86/609/EEC, and had the approval of the Office of the Regional Government of Western Finland. All efforts were made to minimize the pain, discomfort, and number of experimental animals.

The procedure of KA-induced SE was carried out as recently described in detail [18]. Since two age groups (P9 and P21) of Sprague-Dawley rats were used, the procedure applied for each group is described separately. Group 1: P9 rat pups were weighted, and a single dose of KA (1.2 mg/kg) (Tocris Cookson Ltd., Avomouth, UK) was given i.p. Within 20-30 min after the injection, the pups first manifested stiffening, scratching, head nodding, and forelimb clonus with further progression to generalized tonic-clonic seizures within 45-60 min, which continued intermittently for 3-4 h after the KA injection. The mortality of the rat pups was negligible. After the cessation of seizures, the pups were taken back to the cages with their mother, and used for the qPCR and immunocytochemical studies 4 h, 8 h, 24 h and 3 days after the KA injection. Instead of KA injection, P9 control rats received the comparable volume of 0.9% NaCl as given to the KA-treated rats. The KA-treated and their age-matched control rats were separated from their mothers for an equivalent amount of time. Group 2: P21 rats were weighted, and a single dose of KA (7 mg/kg) was given (i.p.). Within 20-30 min, rats first showed scratching, chewing, and myoclonic jerks followed by wet dog shakes, head nodding, forelimb clonus, and rearing and falling. Behavioural signs of generalized tonic-clonic seizures occurred within 45-60 min, which continued intermittently for 3-4 h after the KA injection. The mortality within this age group was negligible as well. Rats were used for the qPCR, immunocytochemical and ELISA studies 4 h, 8 h, 24 h, and 3 days after the KA injection. Instead of KA injection, P21 control rats received the comparable volume of 0.9% NaCl as given to the KA-injected rats, but were otherwise treated as their age-matched treated rats. Rats were visually followed-up for about 2 h after the cessation of behavioural seizure signs, and taken back to their cages. All KA-injected rats of both age groups included in this study manifested the behavioural seizure signs as described above.

### Immunocytochemistry

Immunocytochemistry was performed as previously described in detail for neurofilament proteins with some modifications [18]. Briefly, rats (n = 3 in both age groups of treated and control rats) were deeply anesthetized with 50 mg/kg of pentobarbital, and fixed by transcardiac perfusion with 4% paraformaldehyde in phosphate-buffered saline (PBS, pH 7.4). Brains were rapidly removed, postfixed at +4°C, processed with the antigen retrieval protocol [19], cryoprotected in 30% sucrose in PBS at +4°C, frozen, and kept at -80°C until used. For immunostaining, brains were cryosectioned in 40 μm slices, collected in Tris buffered-saline (TBS, pH 7.4) containing 0.1% Triton X-100, and immediately processed in a free-floating system. First, slices were incubated in a blocking solution (BS) containing 3% bovine serum albumin (BSA), 0.1% Triton X-100 in TBS (pH 7.4), and thereafter with the primary antibodies against Iba1 (1:1000, Wako, Japan) or GFAP (1:1000, Sigma, St Louis, MO, USA) overnight at +4°C in BS. After washings, slices were incubated with a biotin SP-conjugated secondary antibody (1:1000), followed by incubation with the avidin-peroxidase conjugate (Vectastain ABC Kit, Vector Laboratories, Burlingame, CA). Staining was detected using 3,3'-diaminobenzidinetetrahydrochloride (DAB) (Sigma) as a chromogen. In each experiment, KA-treated and control brains were processed simultaneously to minimize the possible interexperimental differences in the staining. Three to four slices, in which the primary antibody was omitted but which were otherwise treated as indicated above, served as negative controls. The immunoreactivity was examined with a Leica DMR microscope (Heerbrugg, Germany) under bright field; pictures were captured using an Olympus U-TV1 X digital camera (Olympus Optical, Tokyo, Japan), and further processed with Adobe Photoshop Elements (version 3.0) and Corel Draw (version 11.0).

In a subset of experiments with the primary antibody against GFAP (1:1000, Sigma), slices were further incubated with a fluorescent secondary antibody Alexa 488 (Invitrogen, Carlsbad, CA, USA), which was diluted (1:1000) in TBS containing 3% BSA and 0.1% Triton X-100. After the staining, slides were dehydrated, coverslipped, and examined with the Leica DMR microscope using the appropriate fluorescence filter. The images were further processed with Corel Draw (version 11.0).

### Real-time quantitative PCR

For qPCR, hippocampi (n = 5-6 in each time-point) were taken from P9 and P21 rats 4 h, 8 h or 24 h after KA injection, and those from saline-injected control groups of the two ages. Additionally, a group of P21 rats, in which hippocampi were prepared 3 days after SE, was used for qPCR, together with their own age-matched control group. Rats were decapitated, hippocampi quickly isolated, immediately frozen in -196°C liquid nitrogen, and stored at -70°C until used. For the experiments, frozen hippocampi were thawed, homogenized with Ultra-Turrax, and the total RNA extraction was carried out with Qiagen RNeasy mini kit (Qiagen, Hilden, Germany) using the protocol supplied by the manufacturer. Total RNA was reverse transcribed to cDNA by M-MuLV Reverse Transcriptase RNase H+ (including RNase inhibitor) (Finnzymes, Espoo, Finland).

PCR was performed using TaqMan One-Step qPCR Master Mix Reagents Kit (Applied Biosystems, Foster City, CA, USA). Amplification was carried out with the ABI 7300 Real-Time PCR System (Applied Biosystems) with a two-step PCR protocol (preincubation of 10 min at +95°C followed by 40 cycles at +95°C for 15 sec, and for 1 min at +60°C). All cytokine primers and probes were designed using the Primer Express software (Applied Biosystems) avoiding contaminating genomic DNA amplification by positioning one of the primers or a probe over the exon/intron boundary. The cytokine cDNA was amplified using the primers and probes as given in Table 1.

**Table 1:** The sequences of primers and probes used to amplify cytokine cDNA

Cytokine	5' primer	3' primer	Probe
**GDNF**	GCCACCATCAAAAGACTGAAAAG	CGGTTCCTCTCTCTTCGAGGA	TCACCAGATAAACAAGCGGCGGCA

**IFN-γ**	TCGAATCGCACCTGATCACTA	GGGTTGTTCACCTCGAACTTG	CATCCTTTTTTGCTTTACTGTTGCTGAGAAG

**IL-1**β	GAAAGACGGCACACCCACC	AAACCGCTTTTCCATCTTCTTCT	TGCAGCTGGAGAGTGTGGATCCCAAAC

**IL-10**	CCCTCTGGATACAGCTGCG	GCTCCACTGCCTTGCTTTTATT	CGCTGTCATCGATTTCTCCCCTGTGA

**MMP-9**	GTATGGTCGTGGCTCTAAACCTG	TCGGCTGTGGTTCAGCTG	CCCAAGGCCTCCAGCCACCAC

**TGF-β1**	TGAGTGGCTGTCTTTTGACGTC	CCTGTATTCCGTCTCCTTGGTT	CTGGAGTTGTCCGGCAGTGGCTGA

**TNF-α**	GACCCTCACACTCAGATCATCTTCT	ACGCTGGCTCAGCCACTC	TAGCCCACGTCGTAGCAAACCACCAA

The probes were labeled with FAM at the 5' end and with TAMRA at the 3' end.

mRNA quantification results were normalized using rat ribosomal 18s RNA (rRNA) (Applied Biosystems) as an endogenous control. Before the study, the stability of rRNA expression in the samples was validated with an experiment in which different concentrations of a sample mRNA were used and rRNA expression was measured. The stability of rRNA also in stress conditions has been previously tested [20]. In all studies, each sample was run in triplicate, and the comparative threshold (CT) method (= ΔΔCT method) was used to examine the relative quantification of the samples (Relative Quantitation computer software, Applied Biosystems). Fold-expression changes were calculated using the equation 2^-ΔΔCT^.

### ELISA

For IL-1β ELISA, three control rats and four KA-injected P21 rats (8 h after injection) were used. Rats were decapitated and hippocampi quickly isolated, placed in ice-cold homogenization buffer containing 50 mM Tris-HCl (pH 7.4), 1% sodium dodecyl sulphate (SDS), 2 mM EDTA, 1 mM phenylmethylsulphonylfluoride (PMSF), and 0.7 mM dithiothreitol, homogenized (Ultra-Turrax T25, Janke and Kunkel, Staufen, Germany), boiled, and centrifuged at +4°C. The supernatants were frozen and stored at -80°C until used. Total protein concentration of the samples was measured using a Lowry-based assay (Bio-Rad, CA, USA). A commercial ELISA kit (Raybiotech, Norcross, GA, USA) was used, and the protocol supplied by the manufacturer was followed. Results are calculated as pg of IL-1β/mg of total protein.

### Western blotting

Western blotting studies were performed as previously described in detail with slight modifications [18]. Briefly, two separate sets of P21 rats, three to four rats at each time point in each set, were used for western blotting studies at 1 and 3 days after KA injection. Different age-matched controls were used for both time-points. Rats were decapitated and hippocampi were rapidly removed and placed in ice-cold homogenization buffer containing 50 mM Tris-HCl, 1% SDS, 2 mM EDTA, 1 mM PMSF, and 0.7 mM DTT (pH 7.4). Thereafter, hippocampi were homogenized, boiled, and centrifuged at +4°C. The supernatants were collected and stored at -70°C until used. Total protein concentration of the samples was measured using the Lowry based Biorad DC Protein assay (Bio-Rad, CA, USA). For SDS-PAGE, equal amounts of protein were applied to each lane of 7.5% acrylamide mini gels, separated by electrophoresis, and transferred to a polyvinylidene fluoride membranes, which were then incubated overnight at +4°C with the monoclonal GFAP primary antibody (Sigma) diluted 1:4500. After that, the membranes were washed and incubated with HRP-conjugated secondary antibody (1:4000) for 1 h at room temperature. The signal was obtained using a chemiluminiscence ECL system (Amersham, Buckinghamshire, UK), and exposed to a film (Hyperfilm ECL, Amersham) that was then developed, and the optical signals were quantified with Image J 1.20s (NIH, USA). The control level was set at 100%, and changes in expression are given as percentage of controls. Western blotting studies were repeated four times with the two sets of rats. Actin (1:4500) (Sigma-Aldrich Inc) was used as a loading control for western blotting.

### Statistical analysis

Statistical significance of differences between groups was analyzed with one-way ANOVA with Tukey's test as a post test for experiments in which there were three or more groups. Differences between the two age groups at the studied time-points were analyzed with two-way ANOVA. Statistical significance of differences in experiments having two groups was analyzed with Student's unpaired t-test. All statistical analyses were performed using the GraphPad Prism software program (version 4.0, GraphPad Software, San Diego, CA, USA). The level of significance was set at p < 0.05.

## Results

### Age-dependent changes in cytokine mRNA expression

mRNA expression of IL-1β, TNF-α, IL-10, MMP-9, GDNF, IFN-γ, and TGF-β1 was investigated 4, 8 and 24 h following KA injection in P9 and P21 rat hippocampi. Additionally, mRNA expression of these cytokines was also studied 3 d after KA injection in P21 rats.

**Figure 1:**
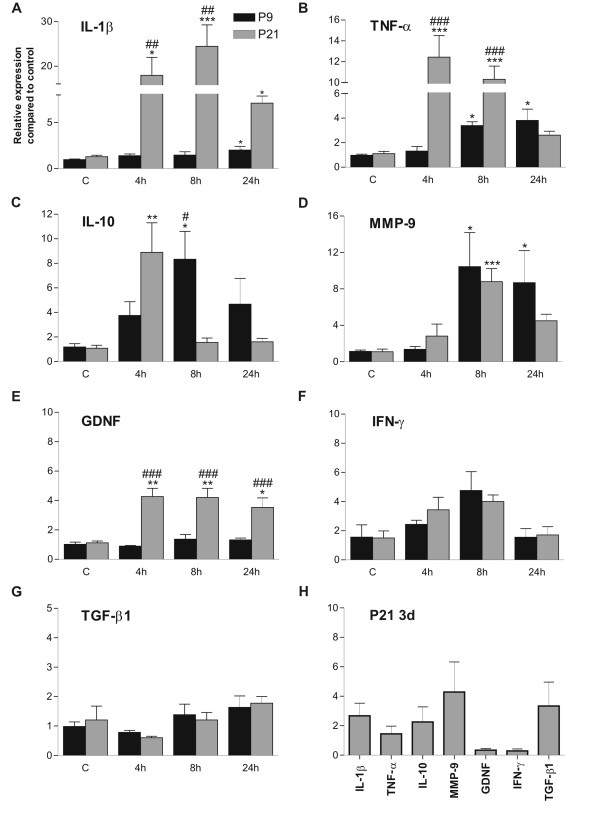
**Cytokine mRNA expression patterns in immature and juvenile hippocampus after KA-induced SE**. Relative quantification of IL-1β (A), TNF-α (B), IL-10 (C), MMP-9 (D), GDNF (E), IFN-γ (F), and TGF-β1 (G) mRNA expression 4, 8 and 24 h after KA-injection in P9 and P21 rat hippocampi, and 3 d after KA-injection in P21 rat hippocampi (H) measured with real-time PCR. Note the significant increases from control levels in both age groups, more pronounced and faster induction of IL-1β, TNF-α and IL-10 mRNA expression in P21 compared to P9 rats, and the increase in GDNF expression only in P21 rats. Note also the return of cytokine mRNA expression in P21 rats to control levels 3 d after KA injection. Data are given as mean ± S.E.M. *p < 0.05, compared to age-matched control (one-way ANOVA); **p < 0.01, compared to age-matched control (one-way ANOVA). ***p < 0.001, compared to age-matched control (one-way ANOVA); ##p < 0.01, compared to the other age at the same time-point (two-way ANOVA); ###p < 0.001, compared to the other age at the same time-point (two-way ANOVA). Abbreviations: C, control; P, postnatal.

IL-1β mRNA expression in P21 rat hippocampi already showed a pronounced increase 4 h after KA injection, peaked at 8 h, when it was almost 25-fold higher compared to that of controls, and still remained significantly higher than in the control group at 24 h (Figure 1A). In contrast, in P9 rats, IL-1β mRNA expression did not significantly change from the control group 4 h or 8 h after KA injection, while at 24 h its expression was slightly but significantly augmented. To confirm the increased expression after SE at the protein level, IL-1β protein expression in the hippocampus of P21 rats was studied with ELISA 8 h after KA injection. The concentration of IL-1β protein was 95.3 ± 7.8 pg/mg (mean ± SEM, n = 3) in the control group, and was significantly (p < 0.05, unpaired t-test) increased to 124.3 ± 10.1 pg/mg (mean ± SEM, n = 4) in the KA-treated group (8 h after injection).

TNF-α mRNA expression closely followed the temporal expression profile of IL-1β (Figure 1B). In P21 rat hippocampi, TNF-α mRNA expression already showed a pronounced increase 4 h after KA injection, at which time it peaked, and thereafter decreased to control levels, its expression not being significantly different from that of control rats at 24 h. In P9 rats, TNF-α mRNA expression was significantly increased over the first 8 h after KA injection, and remained elevated up to 24 h. It is of importance to note that the augmented expression of this cytokine 8 h after KA injection was significantly more pronounced in P21 rats (10-fold) than in P9 rats (3 to 4-fold).

IL-10 mRNA had a somewhat different expression pattern compared to those of IL-1β and TNF-α (Figure 1C). In P21 rats, its expression was significantly increased at 4 h but thereafter it sharply decreased, and at 8 h its expression did not differ from that of the control level. In P9 rats, however, the increase in IL-10 mRNA expression was more gradual, first reaching significance 8 h after SE, and thereafter returning to control levels by 24 h.

The two age groups had more similarities in the MMP-9 mRNA expression patterns (Figure 1D). In both age groups, this expression was significantly increased 8 h after KA. However, in P21 rats the expression decreased to levels comparable to that of their age-matched control group at 24 h, whereas in P9 rats it remained significantly augmented. In P21 rats, GDNF expression was significantly increased at 4 h after KA injection, and remained elevated at 8 h and 24 h (Figure 1E), whereas in P9 rat hippocampi its expression remained unaltered after SE at all time-points studied. In both age groups, the expression of neither IFN-γ (Figure 1F) nor TGF-β1 (Figure 1G) was significantly augmented compared to their respective age-matched controls at any time-point studied.

To study whether the SE-induced increase in expressions of the studied cytokines was prolonged beyond the acute phase, their mRNA expression was also measured 3 d after SE in P21 rats and in their age-matched control group. However, the expression levels of all cytokines studied did not significantly differ from those of controls 3 d after SE (Figure 1H).

### Age-dependent glial cell reactivity after SE

In order to investigate subacute glial cell reactivity after SE in hippocampus, the morphology of microglia and astrocytes was studied with immunohistochemistry 3 d after SE in both age groups. There were no discernible differences in Iba immunostaining in hippocampus between control and KA-treated P9 rats, as microglia retained their resting appearance after KA treatment in both groups (Figurs 2A-D). In contrast, in KA-treated P21 rats, the morphology of microglial cells changed from the resting appereance of the control hippocampi to an active appearance. Activated microglial cells were characterized by enlarged cell bodies and thicker processes in increased numbers (Figurs 2E-H). Microglial activation was detected to some extent throughout the hippocampus, but it is of importance to note that Iba1 immunoreactivity and morphological changes were most prominent in the microglia within the CA1 region. To quantify this, microglia with active appearance in the CA1 region were counted, in a blinded manner, in P21 rats from areas as indicated by the boxes in Figurs 2E and 2G. The number of microglia with an active appearance was 4.5 ± 0.6 (mean ± SEM, n = 4) in the control group and 14.8 ± 0.9 (mean ± SEM, n = 4) in the KA-treated group. This increase was statistically significant (p < 0.0001, Student's unpaired t-test). Also in the amygdala of P21 rats, similar microglial activation was observed 3 d after SE (Figurs 2I-J). In P9 rats, no morphological changes indicating microglial activation were observed in the amygdala (data not shown).

**Figure 2:**
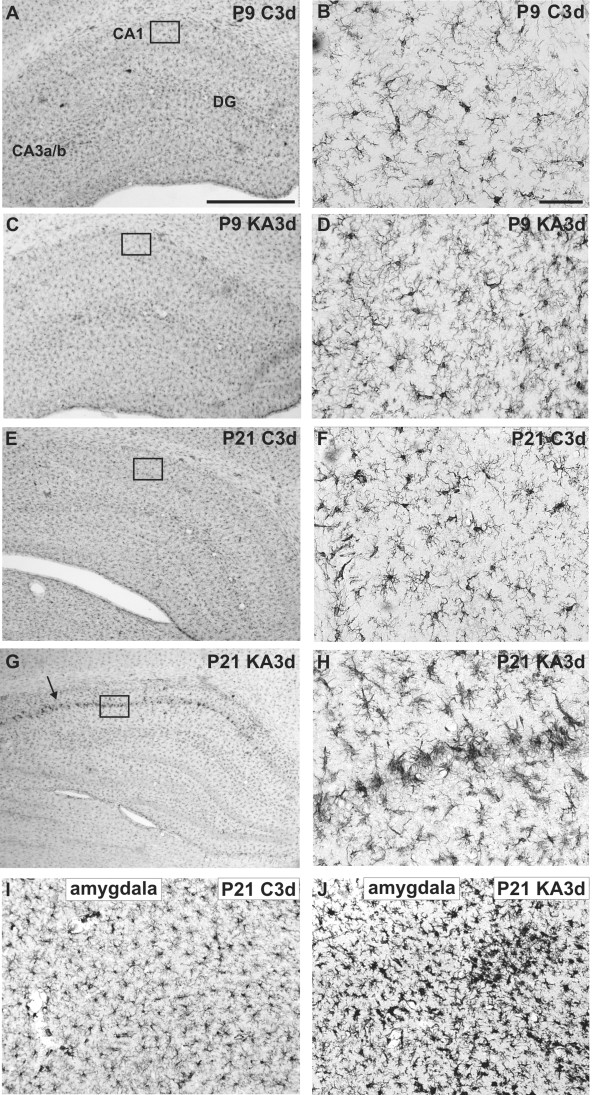
**Microglial morphology in immature and juvenile hippocampus 3 d after SE**. Representative Iba1 immunostaining images of hippocampus for a P9 (A-B) and a P21 (E-F) control rat, and a KA-treated P9 (C-D) and P21 (G-H) rat 3 d after SE, and those of the basomedial amygdala of a control (I) and a KA-treated (J) P21 rat 3 d after SE. The box in the CA1 region in A, C, E, and G is shown with a higher magnification in B, D, F and H, respectively. Note the morphological changes in microglia in the CA1 region (arrow, G) with more pronounced Iba1 immunoreactivity and thickened processes in the KA-treated P21 rat, and the lack of change in P9 rats. Note also the morphological changes of the microglia in amygdala of the P21 rat. Abbreviations: C, control; KA, KA-treated; P, postnatal. Scale bars: 1 mm in A, applicable also for C, E and G; 50 μm in B, applicable also for D, F, H, I and J.

GFAP immunostaining of P9 rat hippocampi did not reveal any changes between controls and KA-treated rats 3 d after SE, including the CA1 region (Figurs 3A-B). In contrast, in KA-treated P21 rats, GFAP immunostaining revealed enlarged cell bodies and thickened processes in astrocytes denoting activated morphology throughout the hippocampus, including the CA1 region, whereas in the control P21 rats astroglial cells in the hippocampus had thin processes and a small cell bodies (Figurs 3C-D). The apparent increase in GFAP expression in P21 rats was confirmed with western blotting, and GFAP protein expression was found to be significantly increased 3 d after SE, but not yet 1 d after SE (Figurs 3E-F).

**Figure 3:**
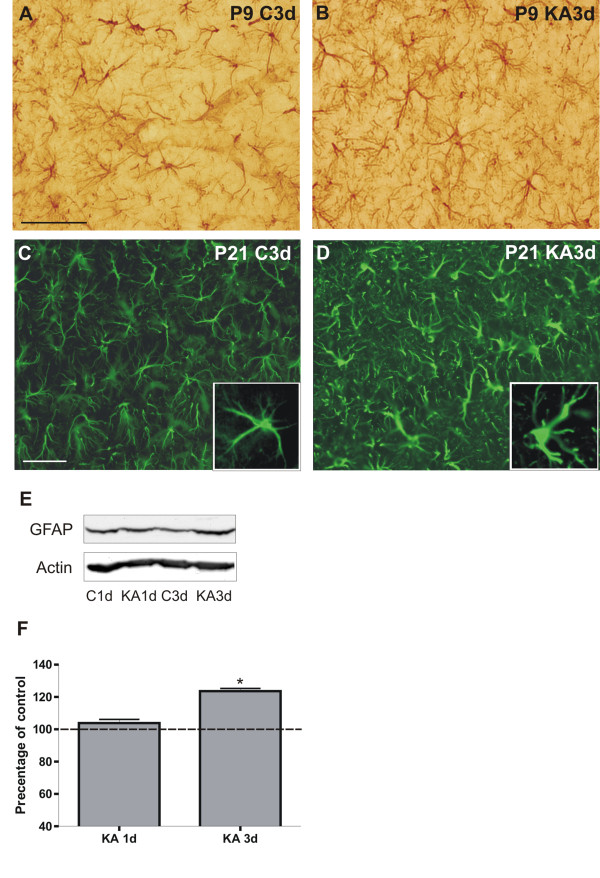
**Astrocyte morphology in immature and juvenile hippocampus 3 d after SE**. Representative images of GFAP immunostainings in the CA1 region of hippocampus for a P9 (A) and a P21 (C) control rat, and a KA-treated P9 (B) and P21 (D) rat 3 d after SE. The box in C and D shows a GFAP-positive cell at higher magnification. Note the prominent morphological changes in astrocytes with thickened, heavily GFAP-positive processes in the KA-treated P21 rat, and the lack of any changes in the P9 rats. A representative western blot (E) and its semi-quantitative analysis (F) of GFAP expression in P21 rat hippocampus in age-matched controls and 1 and 3 d after KA-induced SE. *p < 0.05, compared to the age-matched control group (one-way ANOVA). Abbreviations: C, control; KA, KA-treated; P, postnatal. Scale bars: 50 μm in A, applicable also for B; 50 μm in C, applicable also for D.

## Discussion

The main findings of our study, in which immature and juvenile rats had KA-induced SE, were the following: First, the expression of a number of cytokine mRNAs was induced in both juvenile (P21) and immature (P9) rats after SE; second, the time course and levels of increase in cytokine mRNA expression after SE varied according to the age of rats; third, glial cells exhibited an active appearance in P21 juvenile rats subacutely (3 d) after SE, especially microglia within the CA1 region, while in P9 immature rats glial cells retained their resting appearance. These topics are discussed more thoroughly below.

### Expression of cytokines after SE

It has previously been shown in developing hippocampus that IL-1β mRNA expression is augmented in P15 and P21 rats but not in P9 rats 4 h after KA-induced SE, while TNF-α and IL-6 mRNA expression are increased only in P21 rats [6]. Our recent results are in accordance with these findings and further show that the induction of IL-1β and TNF-α mRNA expression is more prolonged (8 and/or 24 h after SE) in P21 rats and, although to a lesser extent, also occurs in P9 rats. Furthermore, in a previous study in which excitotoxic lesions were induced via NMDA injection in the right sensorimotor cortex of P9 rats, IL-1β and TNF-α protein expression was induced in sensorimotor cortex, corpus callosum, and internal capsule 4 h - 24 h after injection [21]. However, to our knowledge, until now there has been no evidence of any SE-induced cytokine gene expression in P9 rats. Our current results suggest that IL-1β and TNF-α mRNA expression is increased more rapidly and is more pronounced after SE in the more mature juvenile hippocampus compared to a delayed and weak induction in the immature hippocampus. Thus, the time-course and intensity of augmented IL-1β and TNF-α mRNA expression after SE in P21 rat hippocampus resembles that of adult rats [2], which implies that this response is already mature in juvenile rats. It is noteworthy that, compared to controls, the increases in IL-1β protein expression 8 h after KA injection in P21 rats are much lower than the increases in IL-1β mRNA expression at the same time-point. However, the selected time-point probably does not represent the peak in IL-1β protein expression after KA, which is likely to occur later. Furthermore, the result does confirm the tendency of increased mRNA expression to be translated into an increase at the protein level, albeit not on a one-to-one basis. As the mRNA expression levels of many other cytokines were also highly upregulated, it is reasonable to suggest that these are followed by increases in their corresponding protein expressions, although these increases might be significantly lower and occur at later time-points than the mRNA changes.

In addition to neurodegeneration, the classical pro-inflammatory cytokines have been suggested to contribute to epileptogenesis [4]. For example, transgenic mice overexpressing IL-6 showed increased sensitivity to seizures [22], and chronic IL-1β expression has recently been associated with the development of spontaneous limbic seizures after prolonged febrile seizures in P11 rats [23]. Conversely, the IL-1 receptor antagonist (IL-1Ra) mediates anticonvulsant effects in adult rodents in a number of limbic seizure models (i.e. intrahippcompal KA and bicuculline injection, and electrical kindling) [24]. The initial pronounced upregulation of pro-inflammatory cytokine mRNAs seen in juvenile rats in our current study might therefore be an early factor promoting epileptogenesis. While these cytokines can be produced by both glial cell types after seizures, there could be some subtle differences between the glial cell types as to which cytokines they produce, at least in certain seizure models. For example, 12 h after soman-induced seizures in adult rats, IL-1β was observed to be expressed by activated microglia, whereas IL-6 was expressed by astrocytes in the hippocampus, piriform cortex and thalamus [25].

mRNA expression of the anti-inflammatory cytokine IL-10, which has so far been less studied in both immature and mature brain after SE, was augmented in both age groups. In an earlier study in P9 rats, in which excitotoxic brain injury was caused by an intracerebral NMDA injection, IL-10 and its receptor were upregulated in glial cells, suggesting a protective role for IL-10 in excitotoxicity [12]. In our current study, IL-10 expression was increased in concert with the pro-inflammatory cytokines IL-1β and TNF-α, which is in line with the earlier observation that IL-10 expression may be induced simultaneously with pro-inflammatory cytokines in the brain following an insult [26]. Although the pathways of cytokine expression regulation are intricate, the finding that induction of IL-10 mRNA expression was quite moderate and short-lived compared to the more pronounced and prolonged induction of IL-1β and TNF-α mRNA in P21 rats favours the idea that pro-inflammatory cytokine expression might overcome IL-10 expression after SE at this age. In contrast, in P9 rats, IL-10 mRNA expression showed a more pronounced induction than that of IL-1β or TNF-α, which suggests that, at this age, IL-10 might inhibit activated microglial cells and thus suppress expression of pro-inflammatory cytokines after SE more effectively than in P21 animals. Furthermore, microglial cells expressing IL-10 have been shown to attenuate neurodegeneration in hippocampal cultures when apopotosis is promoted with NMDA [27]. This enables the hypothesis that microglia might self-limit their response via negative feedback by producing IL-10 and thus can have either pro-inflammatory or anti-inflammatory functions, depending on circumstances.

Also MMP-9 mRNA expression increased in both age groups after SE. In *in vitro *conditions, MMP-9 has been shown to be produced by activated microglia [28], and cytokines are proposed to regulate MMP-9 activity in astrocytes [29]. In an earlier study, increased MMP-9 activity was observed 8 h after KA-induced seizures in adult rats, and this was linked with neuronal death as confirmed in KA-treated organotypic hippocampal slice cultures (prepared from P11 rats and cultured for 14 days) [30]. In our current study, MMP-9 mRNA expression increased in both P21 and P9 rats and, as previous studies have shown that P9 rats do not exhibit any detectable neuronal damage from KA-induced SE [6,8], this suggests that MMP-9 mRNA upregulation after SE alone is not sufficient to induce neuronal damage in the P21 rat hippocampus. MMP-9 might have a more central role in epileptogenesis, as MMP-9 knockout mice show lower sensitivity to epileptogenesis after pentylenetetrazole kindling-induced seizures, while the sensitivity of transgenic rats overexpressing MMP-9 is higher [31].

GDNF mRNA expression is augmented in the adult rat hippocampus 6-24 h after KA-induced SE [16]. In our study in developing rats, GDNF was the only studied cytokine whose mRNA was increased exclusively in P21 rats after SE. In another earlier study, in which SE was induced with KA in adult rats, adenoviral-vector-delivered GDNF, introduced before KA injection, increased Bcl-2 expression and reduced the number of apoptotic cells in the CA3 and dentate gyrus (DG) regions of the hippocampus compared to the rats, which did not receive GDNF [32]. This suggests that GDNF has a neuroprotective role in KA-induced SE. Furthermore, GDNF is proposed to suppress seizures in temporal lope epilepsy models (e.g. kindling) in adult rats [33]. Our results indicate that GDNF upregulation after SE is a mechanism that has reached an adult level of maturity in P21 rats.

The expression of IFN-γ and TGF-β1 mRNA did not significantly change after SE in either age group. In a recent study in adult rats, IFN-γ mRNA expression was increased in astrocytes of the hippocampus 24 h after lithium-pilocarpine-induced SE while the expression of its receptor was upregulated in neurons and, furthermore, neutralization of the IFN-γ receptor aggravated injury suggesting a protective role for IFN-γ [34]. Also, the expression of TGF-β1 mRNA has been shown to be increased in response to KA-induced SE in microglial cells in the hippocampus of adult rats [35]. However, TGF-β1 has recently been suggested to contribute to epileptogenesis induced by albumin after blood-brain barrier breakdown in adult rats [36]. Our current findings suggest that these mechanisms, which are activated by SE in the adult hippocampus, are still underdeveloped in the juvenile P21 rat hippocampus.

Our present results together with earlier findings of other groups suggest that the more mature innate immunity system elicits a fulminant inflammatory response after SE, in which the pro-inflammatory reaction is marked. In contrast, the immature system evokes only a mild pro-inflammatory response, but a marked anti-inflammatory reaction. We propose that in the immature brain either the ability to induce acute inflammatory reaction is underdeveloped or its emphasis is on anti-inflammatory properties. Moreover, the inflammatory reaction in developing hippocampus is likely to at least partly contribute to age-dependent susceptibility to SE-induced neuronal injury and recurrence of seizures. However, other factors, such as molecular and functional differences in receptors and ion channels between the immature and adult rodent brain, may be of equal or even greater importance in determining changes in the seizure threshold [37].

### Glial cell activation after SE at the acute and the subacute phase

Glial cells, i.e. microglia and astrocytes, are age-dependently activated (in P21 and P15, but not in P9) in the postnatal rat hippocampus up to 24 h after KA-induced SE [6]. Additionally, short-term microglial (a few days) and long-term (beyond 40 days) astrocytic activation have been observed after KA-induced seizures in P15 rats, and this has been proposed to contribute to neuronal injury, neurobehavioral impairment, and increased susceptibility to seizures [38]. Our results further address this and show that also subacutely, 3 days after seizures, both types of glial cells remain activated in P21 rats and that glial cells remain inactivated in P9 rats also at this subacute phase. Furthermore, astrocyte activity seems to increase over time after SE in P21 rats, since GFAP expression was now significantly increased 3 d after SE, but not yet 1 d after SE. This further supports the notion that the more mature brain elicits a more prominent inflammatory response after SE, which may be in part due to a diminished ability of glial cells to self-limit their response. Additionally, it is of importance to note that the most pronounced subacute microglial activation was localized to the CA1 pyramidal cell layer in P21 rats after KA treatment, which is the region damaged by KA-induced SE in rats of this age [8]. In contrast, an earlier study has shown that microglia are equally activated in all hippocampal regions 4 h and 24 h after onset of SE in P21 rats [6]. Our results therefore suggest that the generalized microglial response in hippocampus seems to be gradually and specifically concentrated on the damaged CA1 region at the subacute phase in P21 rats, whereas the astrocytic response remains ubiquitous in the hippocampus. Furthermore, our recent transcriptomic study indicated pronounced upregulation of the GFAP gene in the CA1 region together with neuronal damage in P21 rats 7 days after SE [39], which denotes long-term astrocyte activation specifically in the damaged area.

In contrast to prolonged glial cell reaction, increased expression of cytokine mRNAs is transient and, as our results show, at three days after SE in P21 rat hippocampi, cytokine mRNA expression has returned to control levels. This suggests that activated glial cells in juvenile hippocampus may have functions other than cytokine production during the subacute phase. Indeed, microglial response has been shown to be diverse after their activation, and can include both neurodegenerative and neuroprotective functions [40]. The induction of COX-2 expression in adult and P21 rat brain has been shown to be transient as well, since its expression returns to the control level within three days after SE [6,41]. This further suggests that after the initial acute phase (which seems to last up to 24 h - 3 d), there is a change in the inflammatory response provoked by KA-induced SE in juvenile hippocampus, with attenuation of the pronounced, and perhaps exaggerated, expression of inflammatory mediators and modulation of microglial reactivity from widespread activation to a targeted response in the damaged area.

In our study, microglial activation was now detected in P21 rats 3 d after SE also in the basomedial amygdala, which together with the hippocampus is a part of the limbic system and suffers neuronal damage after KA-induced SE [6]. Furthermore, the expression of COX-2 and c-Fos has been shown to be markedly increased in P21 rats after SE in amygdala, this again in tandem with hippocampus [8,42]. Thus, after the acute phase post-SE, the inflammatory response at the subacute phase seems to be more accurately targeted to damaged areas of limbic structures in juvenile rat brain.

## Conclusions

In conclusion, SE transiently induced mRNA expression of a number of cytokines in both immature and more mature juvenile rat hippocampus. In general, the increases in cytokine mRNA expressions were more pronounced and rapid in juvenile brain than in immature brain. Glial cells still exhibited an active appearance subacutely after SE in juvenile hippocampus while retaining their resting appearance in immature hippocampus. In juvenile hippocampus, the transient augmentation of cytokine mRNA expression after SE, in contrast to the persistence of glial cell reaction including region-specific microglial activity at the subacute phase, suggests a regulated shift from a fulminant and general initial reaction towards a more moderate and precisely targeted response.

## Competing interests

The authors declare that they have no competing interests.

## Authors' contributions

JTJ conceived and designed the study, carried out qPCR, ELISA and immunohistochemical experiments, performed statistical analyzes, and drafted and revised the manuscript. AP carried out most of the qPCR and some of the immunochemical experiments, and FRL carried out the animal treatments. SR designed and supplied the primers and probes used in PCR experiments. IEH supervised the study, and participated in designing the study, and in drafting and revising the manuscript. All authors read and approved the final manuscript.
